# How much is too much?—Influence of X-ray dose on root growth of faba bean (*Vicia faba*) and barley (*Hordeum vulgare*)

**DOI:** 10.1371/journal.pone.0193669

**Published:** 2018-03-26

**Authors:** Sebastian R. G. A. Blaser, Steffen Schlüter, Doris Vetterlein

**Affiliations:** 1 Helmholtz-Centre for Environmental Research GmbH–UFZ; Department of Soil System Science, Halle (Saale), Germany; 2 Martin-Luther-University Halle-Wittenberg; Institute of Agricultural and Nutritional Sciences, Halle (Saale), Germany; Iwate University, JAPAN

## Abstract

X-ray CT is a powerful technology to study root growth in soil *in-situ*. Root systems can be studied in its true 3D geometry over time. Hence, the same plant can be scanned multiple times during development. A downside is the potential of X-rays to interfere with biological processes and therefore plant growth. The aim of this study is to evaluate the influence of cumulative X-ray dose on *Vicia faba* and *Hordeum vulgare* during a growth period of 17 days. One control treatment without X-ray scanning was compared to two treatments being scanned every two and four days, respectively. Scanned treatments received a maximum cumulative dose of less than 8 Gy. Plant species differed in their susceptibility to X-ray dose. For *Vicia faba*, mean total root length was reduced significantly. Leave growth was reduced as well. Number and length of second order laterals was reduced significantly, as well as length of first order laterals. *Hordeum vulgare* showed no negative impact of X-ray dose on any of the root parameters. Large differences between the two species investigated were detected in respect to susceptibility to X-ray dose. Results indicate that for X-ray CT studies involving temporal resolution a control treatment without scanning is required.

## Introduction

CT is a very powerful technology to study root growth in soil *in-situ*. Due to its non-invasive nature, the same plant can be scanned multiple times during plant development to study root growth dynamics. By this, plants receive a cumulated dose of X-ray radiation. Depending on plant species, growing period, scanning frequency (and duration), scanning settings (energy and current, but also source to sample distance) and used filters, this dose might be high enough to influence root growth. Early studies have shown that X-ray radiation can have a destructive influence on auxin and affects meristematic cells, as cytological changes like mitotic cycle delay, chromosome aberrations or loss of proliferative capacity were observed [1–8].

Studies using X-ray tomography to analyse root growth over time, show large differences in total X-ray dose received by plants [9–15]. Several authors try to keep the dose very low, being aware that the dose may affect root growth [16] or use older plants (e.g. [11, 17]), assuming a reduced sensitivity of older plants to radiation exposure. Only two recent studies working with X-ray CT included a control treatment without scanning to evaluate potential harmful effects of X-ray dose [10, 18]–both have been working with cereals.

Therefore, as summarised in a recent review by Zappala *et al*. [18], there is a lack of information on the influence of the method itself on the parameter in question. The dose of 33 Gy which according to the conclusion of Zappala *et al*. [18] should not significantly influence growth provides a first orientation. In the present paper we will show that this threshold does not hold true for all plant species.

It is expected that apart from X-ray CT system specific parameters (energy settings, filter and distance) and the soil used, also plant age and especially root system and root architecture patterns are important. The early and comprehensive work of Johnson [3] on seventy species (35 families) of flowering plants and their exposure to X-rays gives a broad overview, but is difficult to interpret, as CT settings, number of replicates and plant age differ strongly and almost exclusively aboveground parts of the plants were considered for evaluation of the results. Johnson [3] grouped the studied families in three divisions, being i) apparently unaffected, ii) slightly affected and iii) species noticeably affected by the X-rays. All plant species investigated by Johnson [3] were dicotyledonous. Hence, no comparison between dicots and monocots can be derived from these results. Moreover, these results show that even within dicots, large differences exist regarding sensitivity against radiation.

Apart from the overall influence of cumulative X-ray dose, the question is whether there is a specific effect on initiation of root primordia and hence lateral root formation. This would lead to problems when X-ray CT is used to visualize and quantify root system changes in response to different stimuli like nutrient patches, water distribution or mechanical impedance.

The aim of this study was to evaluate the influence of cumulated X-ray dose (< 8 Gy) on two plant species in a given experimental setup. One species was faba bean (*Vicia faba*), a tap rooted dicot plant with a rather coarse root system, with large root diameters also for first and second order laterals. Due to this root phenotype *Vicia faba* is particularly suitable for X-ray CT studies, overcoming in part the trade-off between sample size and image resolution. The second plant species was barley (*Hordeum vulgare*), a monocot with an adventitious root system, having smaller root diameters than *Vicia faba*. One control treatment was established in both experiments and not scanned until one day before harvest and is compared to the two treatments being scanned every two and four days, respectively. These frequencies are typical for X-ray studies on root growth dynamics under controlled conditions.

## Material and methods

### Soil preparation, plant material and growth conditions

Acrylic columns (250 mm height, 35 mm radius, 5 mm wall thickness) were filled with a sieved (< 2 mm) and homogenised silty clay loam and packed to a bulk density of 1.2 g cm^-3^. For more details of the soil, see Vetterlein *et al*. [19]. The soil was N-fertilised with five urea-granules per sample, containing a nitrification inhibitor (ALZON46, SWK Piesteritz) at a rate of 100 mg N kg^-1^ soil in one layer 6 cm below soil surface. This was done as these granules could be visualised in the CT and served as a spatial orientation. The soil was initially watered by capillary rise with 239 ml distilled water, resulting in a water content of about 27 vol.% (= pF 2.65). Three treatments with five replications were set up for *Vicia faba*. Three treatments with at least six replications (two of the treatments with seven replications) were set up for *Hordeum vulgare*. Each column was placed on a weighing cell to record total transpiration and to enable keeping water content as stable as possible. Soil columns were wrapped with aluminium foil to prevent algae growth. Seeds of faba bean (*Vicia faba* L., cv. ‘Fuego’) and barley (*Hordeum vulgare*, cv. ‘Marta’) were surface sterilised with H_2_O_2_ (10%) and soaked in saturated CaSO_4_ for four hours before one seed was placed per column about 1.5 cm below soil surface. Coarse gravel was placed on top of the soil to reduce evaporation from soil surface. Plants were grown for 17 days in a climate chamber under controlled conditions (12/12 h day/night cycles at 19 and 16°C, respectively. Photosynthetic active radiation was 350 μmol m^-2^ s^-1^).

### CT scanning configuration and image analysis

X-ray tomography was performed with an industrial μCT (X-TEK XTH 225, Nikon Metrology) with 140 kV, 286 μA (equals 40 Watts) and 500 ms exposure time. Each scan was performed with 1000 projections and one frame per projection, resulting in an exposure time of 8.5 minutes per scan. A copper filter with 0.5 mm thickness was used to reduce beam hardening artefacts. Distance between X-ray source and sample was about 13 cm. The spatial resolution of the X-ray tomogram is 40 μm. The calculated dose rate for these settings is 480 R h ^-1^ (calculated with the Rad Pro Calculator for Desktop PCs Version 3.26 from http://www.radprocalculator.com/RadProDownloads.aspx). This equals 421 rad h^-1^ (calculated with the conversion factor 1 R = 0.877 rad (to air) from Cember and Johnson [20]), equal 4.2 Gy h^-1^. The Rad Pro Calculator tool calculates the dose in air, not in soil. Soil properties (or growth media in general) and soil moisture is neglected in the calculator. Therefore the comparability of different doses is only warranted for the external dose at the wall of the soil container, but not for the dose that reaches the plant roots in the soil column. In the course of the paper, the calculated doses therefore refer to the *maximum* dose acting on the container wall.

The first tomograms were performed 4 days after planting (DAP) for both treatments. The control (C) treatment was only scanned on the last day before harvest for comparison. The other two treatments were scanned every two (‘frequent scanning’ (FS)) and every four days (‘moderate scanning’ (MS)), respectively. From 6 DAP onwards, two tomograms, one above the other, had to be conducted to capture the major part of the root system (equivalent to the top 14 cm of the soil column). The remaining part of the soil column was not shielded during the other scans. Moreover a small overlap between both scans was required to enable the concatenation of both scans per sample for subsequent image processing. In this 1–2 cm large region the received dose for the roots is expected to be even higher. Moreover, the remaining part outside the region of interest during a scan is expected to receive further radiation from the X-ray beam and additional scattered radiation. During the growing period of 17 days, this scanning procedure results in 7 scans for the treatment ‘moderate scanning’ and 13 scans for the treatment ‘frequent scanning’. The cumulative scanning times and doses per sample are 60 and 111 minutes, or 4.2 and 7.8 Gy, respectively.

All X-ray scans were performed during the night phase in the climate chamber. The X-ray tomograph and the climate chamber are located next door to each other.

For *Vicia faba*, a detailed analysis of CT-images was conducted. Main points are stated here, for more details see supporting information, S1 Method. Raw data was filtered to reduce image noise. Root systems were segmented with semi-automated region growing in VG Studio Max 2.1. Based on the idea of Flavel *et al*. [10], binarised root systems were skeletonised and analysed with the plugin BoneJ [21] in Fiji [22]. The resulting information was used to quantify root length and to distinguish between tap root, first and second order lateral roots. CT-images of segmented roots from consecutive time steps were spatially aligned with the elastix image registration software [23, 24].

Due to the small diameters of *Hordeum vulgare* roots, short scanning times and therefore weak image contrast between root tissue and water-filled pores, analysis of *Hordeum vulgare* roots was not possible in the same way as for roots of *Vicia faba*. Therefore, for *Hordeum vulgare* only the results for the washed out roots analysed with WinRHIZO are presented.

### Shoot and root analysis

At 17 DAP, the plants were harvested. Shoot fresh weight and leaf area were measured directly after harvest. Roots were washed out and analysed with WinRHIZO 2009b (Regent Instruments, Canada). WinRHIZO analysis was performed for total root length and three functional diameter classes. For *Vicia faba*, thresholds were set at 0.75 and 1.25 mm with the intention to separate first and second order laterals from tap roots, based on root diameters. The smallest class below 0.1 mm was discarded, as it is error-prone due to root hairs causing misclassification.

For *Hordeum vulgare*, thresholds were set at 0.20 and 0.50 mm with the intention to separate first and second order laterals from seminal roots based on root diameters. The smallest class below 0.05 mm was discarded, as it is error-prone due to root hairs causing misclassification.

Leaf area was also measured by WinRHIZO 2009b. Plant samples were oven dried afterwards at 65°C for 48 hours.

### Statistics

Statistics (T-test and Scheffé post-hoc-test) were performed with SPSS 22 (IBM) at P<0.05. Standard errors are given in all figures as error bars.

## Results

### 1. *Vicia faba*

**Fig 1:**
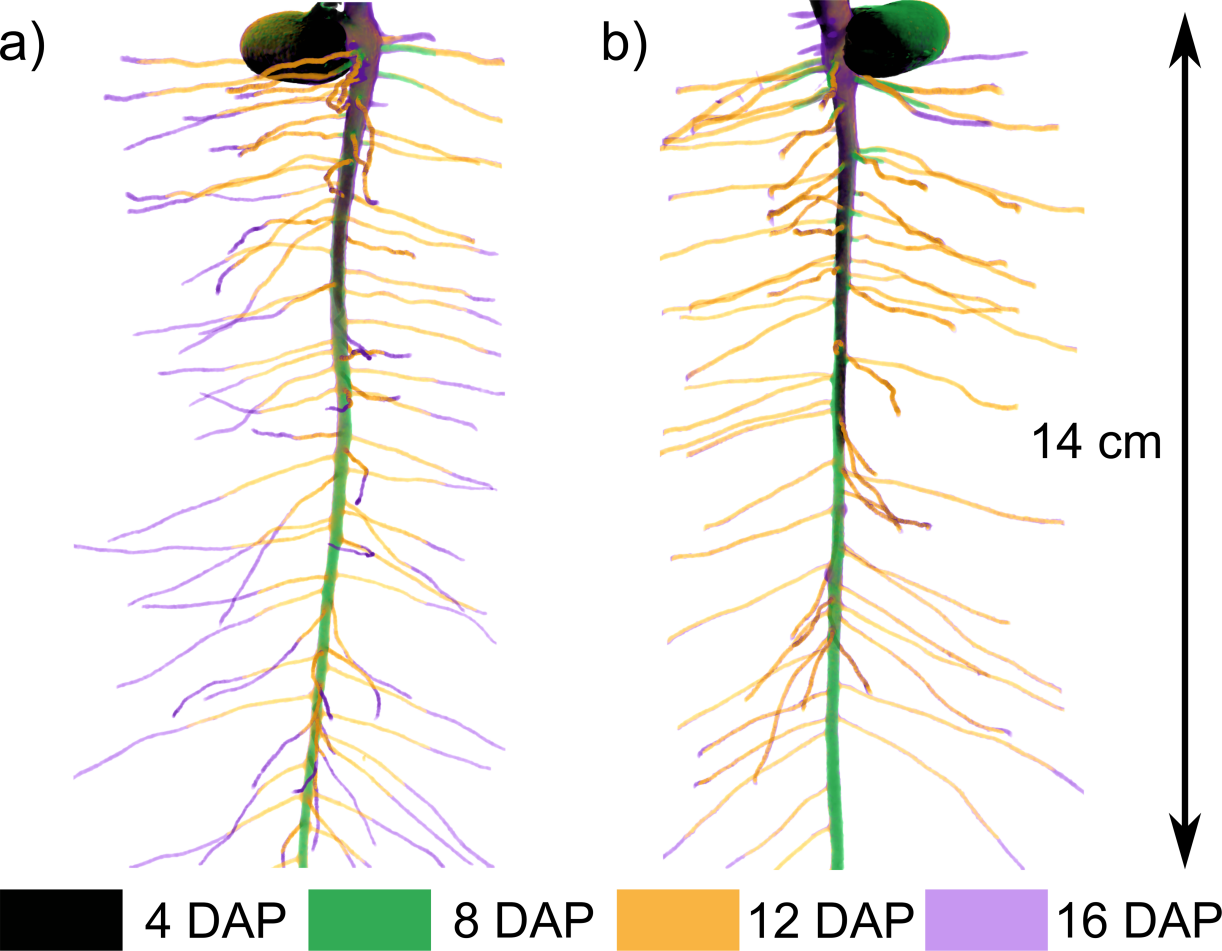
Time series of root system development of *Vicia faba*, acquired by X-ray CT. Representative 2D projections for both scanned treatments: a) frequent scanning (FS) and b) moderate scanning (MS). Root age is colour coded for 4 (black), 8 (green), 12 (orange) and 16 (purple) days after planting (DAP). Changes in position are also recorded; this is the reason for the green shade at the seed in b). Secondary thickening can also be seen by the purple shade around the upper part of both tap roots. Illustrating videos for those two samples are available in the supporting information (S1 and S2 Videos).

Root growth development can be visualised by X-ray CT with a very high degree of detail for *Vicia faba* (Fig 1 and S1–S5 Figs for all replicates). The spatial resolution of 40 μm is sufficient to visualise all root orders of *Vicia faba*, emerging during the first 16 days of root system development, except for those growing along the container walls. It was thus possible to follow initiation of first order laterals starting to appear at day 8. Second order laterals were only observed at day 16.

#### CT data

**Fig 2:**
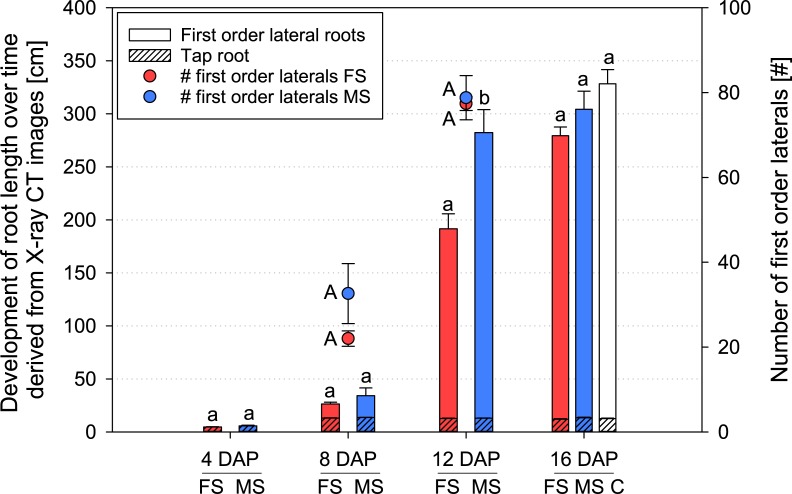
Root length (bars) and number of first order laterals (circles) of *Vicia faba* over time measured by X-ray CT; FS = frequent scanning; MS = moderate scanning; C = no scanning (control); data is only shown for time steps when both treatments were scanned. Small letters refer to significant differences in root length and capital letters in number of laterals. Standard errors are given as error bars.

As expected, the mean length of *Vicia faba* taproots did not differ among the treatments. Likewise, number of first order laterals showed no significant difference between both scanned treatments (Fig 2). For length of laterals, this is not the case. At 8 DAP the difference is not yet significant, but at 12 DAP the first order lateral roots of the ‘moderate scanning’ (MS) treatment are significantly longer than those scanned every second day (frequent scanning; ‘FS’, Fig 2). The difference between both treatments is 66.4%, very close to the overall difference in total root length at the end of the experiment, measured by WinRHIZO (66.7%). For the last point in time, again no significant differences were observed. Root length captured by X-ray CT at day 16 accounted for only 27–47% of total root length measured by WinRHIZO analysis at day 17. Roots growing below the CT’s field of view (14–22 cm depth of soil columns) and those, growing at the container wall could not be observed.

**Fig 3:**
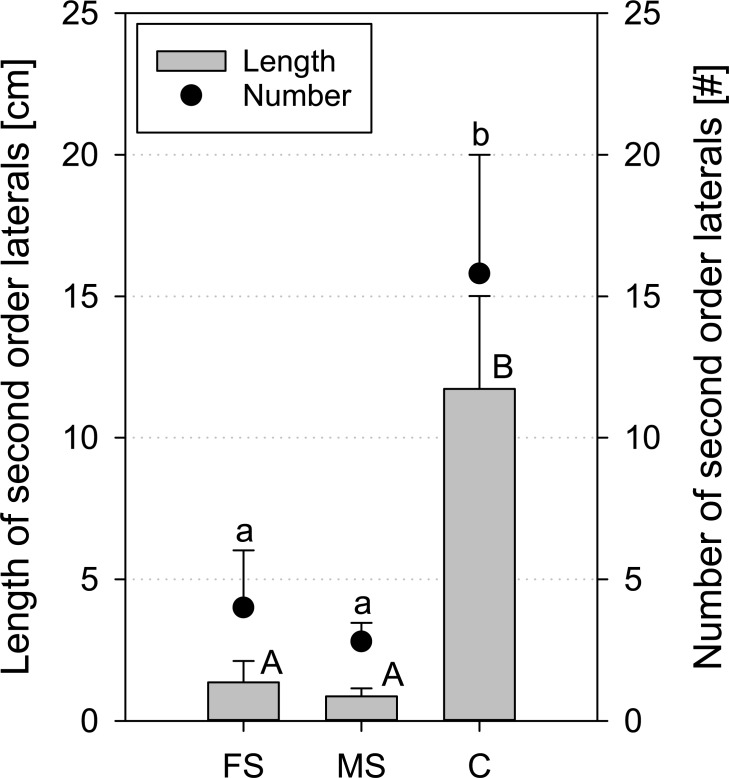
Length and number of second order laterals of *Vicia faba* measured with X-ray CT at 16 DAP; standard errors are given as error bars.

Apart from taproots and first order laterals, also number and length of most second order laterals was determined. This analysis was made for the data at 16 DAP, one day before harvest, comprising all three treatments (Fig 3). Both scanned treatments showed very few second order laterals (4.0 ± 2.0 for frequent scanning and 2.8 ± 0.7 for moderate scanning) with a small total length of 1.4 (± 0.8) and 0.9 (± 0.3) cm for frequent and moderate scanning, respectively. A high standard error for treatment ‘frequent scanning’ arose from one plant, having more second order laterals than all other replicates. For the control treatment, length and number of second order laterals were significantly larger (Fig 3).

**Fig 4:**
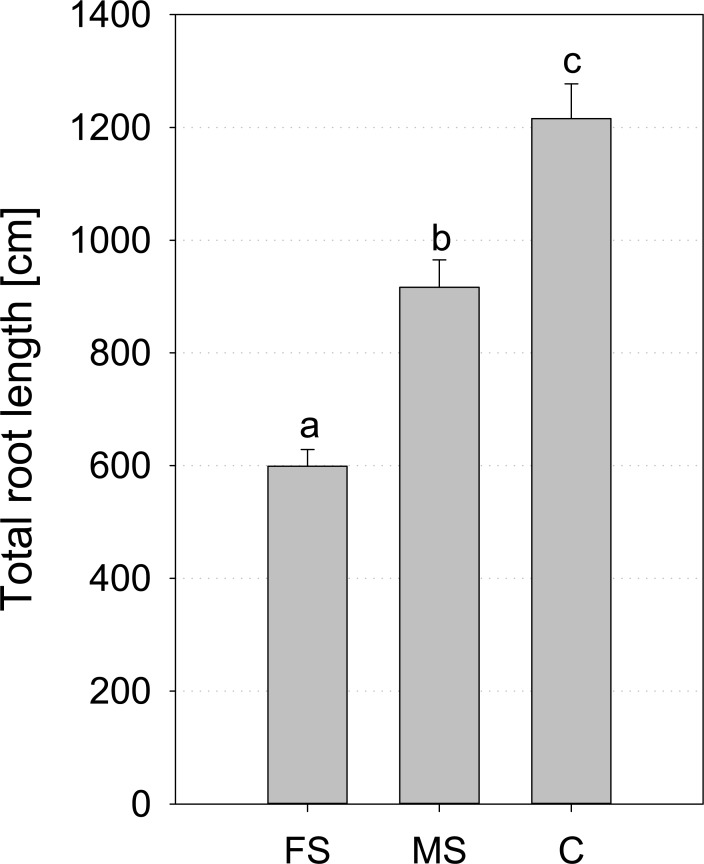
Mean total root length of *Vicia faba* at the end of the experiment (17 DAP) measured with WinRHIZO; FS = frequent scanning; MS = moderate scanning; C = control (no scanning); standard errors are given as error bars.

Total root length at 16 DAP was reduced by X-ray scanning with high and moderate frequency (Fig 2). This result is confirmed by WinRHIZO analysis (Fig 4). Reduction was significant in comparison to control, but also both scanned treatments differed significantly. The mean reduction of scanned treatments in comparison to control is 25% for moderate scanning and 51% for frequent scanning.

**Fig 5:**
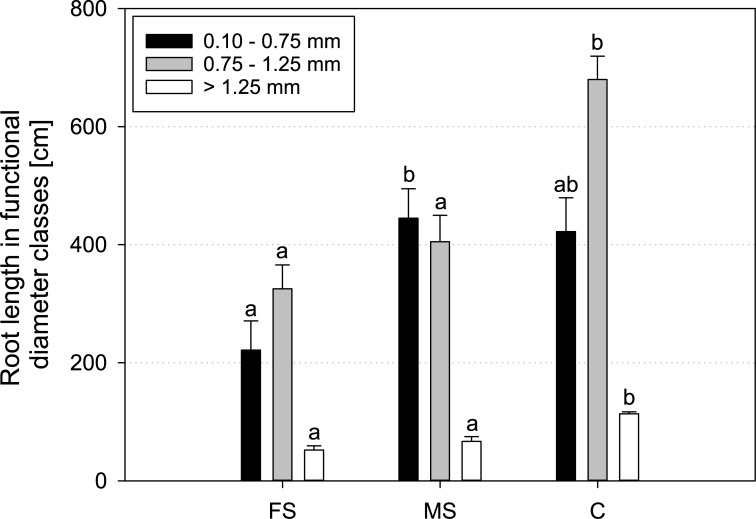
Mean root length in functional diameter classes of *Vicia faba* measured with WinRHIZO at the end of the experiment (17 DAP); FS = frequent scanning, MS = moderate scanning, C = control; smallest diameter class < 0.10 mm is discarded due to distorted values by influence of root hairs; standard errors are given as error bars.

Functional diameter classes, defined in WinRHIZO to distinguish between tap root, first and second order laterals, did not show clear differences between treatments (Fig 5). Length of first order laterals was significantly higher in the control, compared to both scanned treatments. However, root length in the smallest diameter class–which was supposed to represent second order laterals–did not match with the results from X-ray CT. Comparison of root length in this class obtained with both methods clearly indicates that threshold setting with WinRHIZO is not an adequate metric to derive root orders, because of similar root diameters for first and second order laterals and the conical shape of roots towards the root tip.

**Fig 6:**
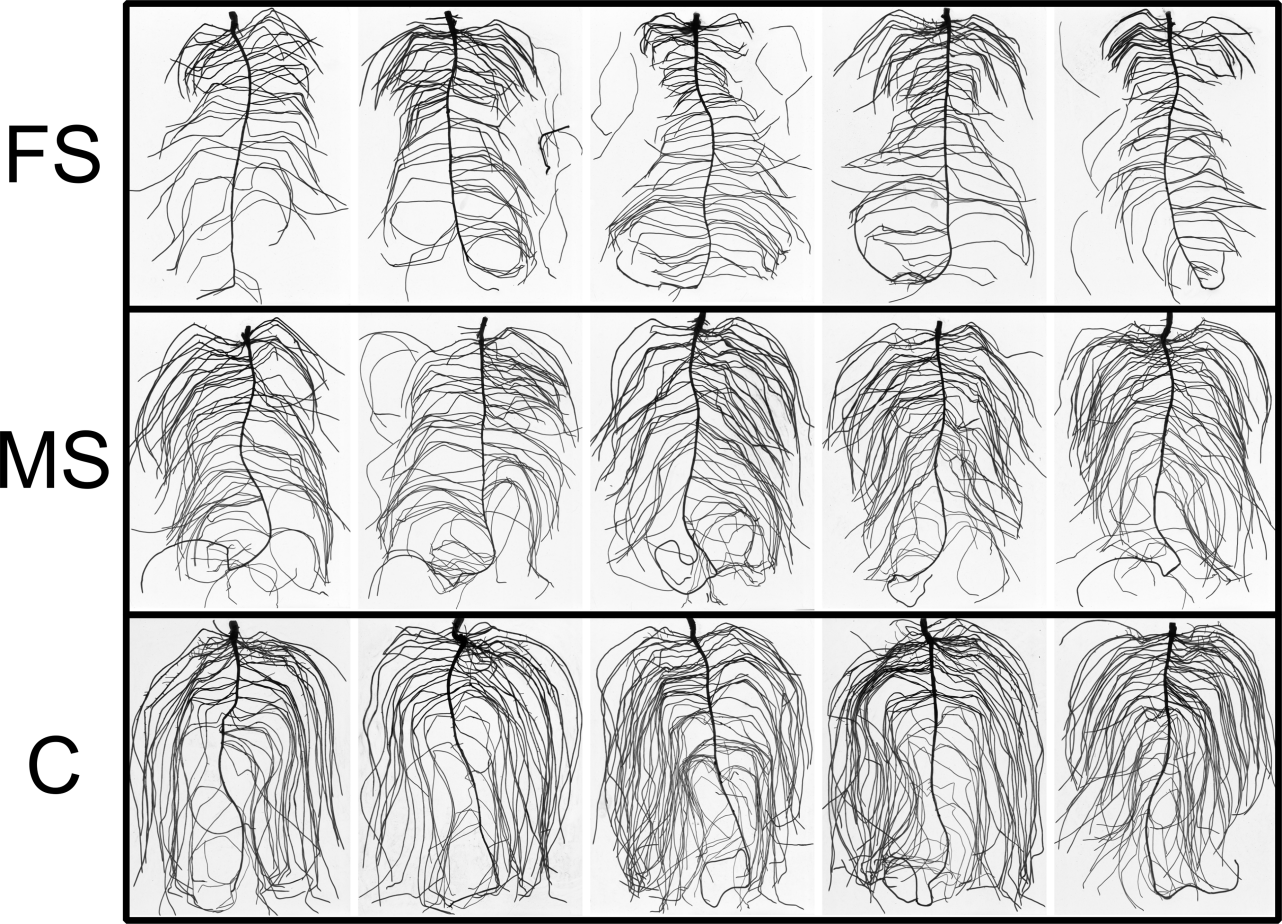
Washed out root systems from *Vicia faba* of all treatments (first row: frequent scanning (scanning every second day), middle row: moderate scanning (scanning every fourth day), bottom row: control without scanning) at the end of the experiment (17 DAP).

Changes in root growth are not only apparent from root length data but also from visual inspection of the root system after destructive sampling at the end of the experiment. Root systems from control treatment show many elongated lateral roots with several lateral roots of second order at the oldest laterals (Fig 6). Most of the root systems from treatment ‘moderate scanning’ show smaller elongation of first order lateral roots and still some 2^nd^ order laterals. For the treatment receiving the highest X-ray dose (‘frequent scanning’), root system architecture reveals short laterals, especially in the upper third of the root system. This part of the root system received the highest total dose as it has been in the focus of scanning from day four after planting and also received further X-ray radiation during each scan of the lower part, as it was not shielded during the other scans. Overall, laterals are very short compared to the other treatments. Additionally, only very few 2^nd^ order laterals can be found for most of the replicates. Moreover, roots were more brownish and brittle for the treatment of frequent scanning.

#### Leaf area and shoot weight

**Fig 7:**
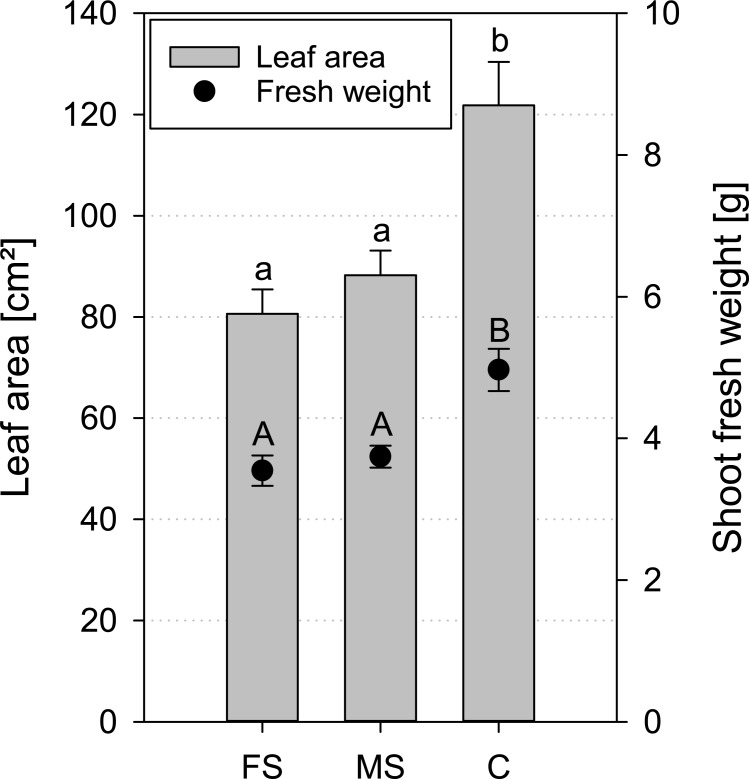
Mean leaf area and fresh weight of *Vicia faba* for frequent scanning (FS), moderate scanning (MS) and control treatment (C) at the end of the experiment (17 DAP); standard errors are given as error bars.

Mean fresh weight and mean leaf area of *Vicia faba* plants were significantly lower for both scanned treatments in comparison to the control. On average, mean shoot fresh weight was reduced by 25–29%, mean leaf area by 41–51% for both scanned treatments (Fig 7).

### 2. *Hordeum vulgare*

**Fig 8:**
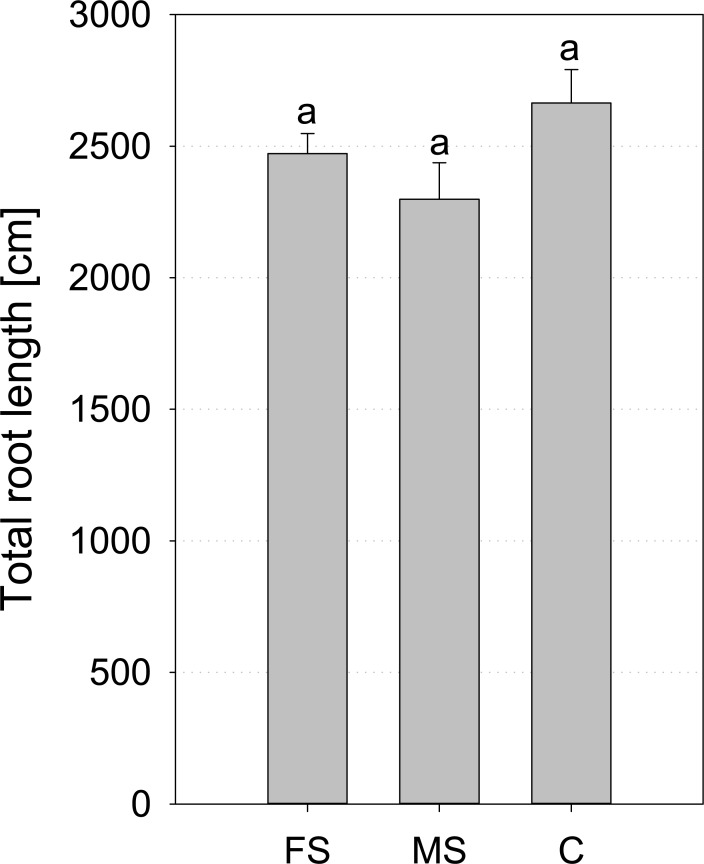
Mean total root length of *Hordeum vulgare* measured with WinRHIZO at the end of the experiment (17 DAP); FS = frequent scanning, MS = moderate scanning, C = control; standard errors are given as error bars.

For *Hordeum vulgare*, analysis of CT data was not possible due to very small root diameters and insufficient image quality in terms of image resolution and contrast. Hence, only washed out roots (S6 Fig) were analysed with WinRHIZO at the end of the experiment. Results show, that both treatments of X-ray radiation had no influence on root growth for *Hordeum vulgare* in comparison to the un-scanned control treatment (Fig 8).

**Fig 9:**
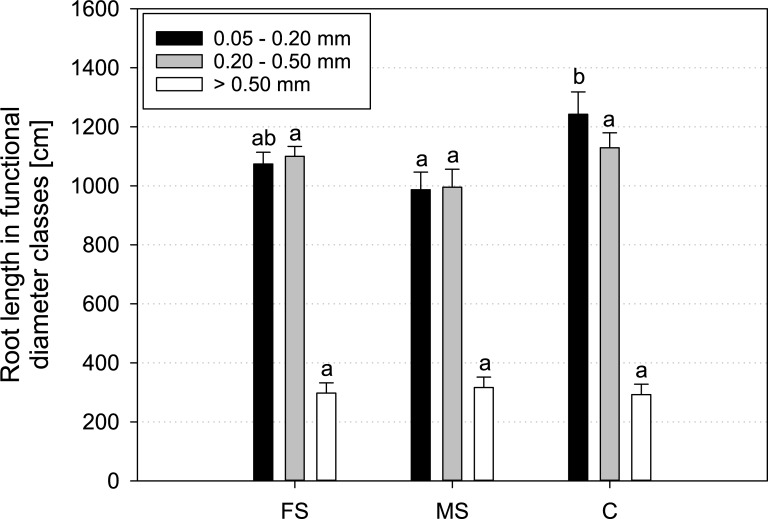
Mean root length of *Hordeum vulgare* in functional diameter classes measured with WinRHIZO at the end of the experiment (17 DAP); FS = frequent scanning, MS = moderate scanning, C = control; smallest diameter class < 0.05 mm is discarded due to distorted values by influence of root hairs; standard errors are given as error bars.

Like for *Vicia faba*, functional diameter classes were also chosen for *Hordeum vulgare*. Results are very similar for all treatments. There is a tendency towards a higher length of finest roots in the control treatment, but the absolute differences are small (Fig 9).

**Fig 10:**
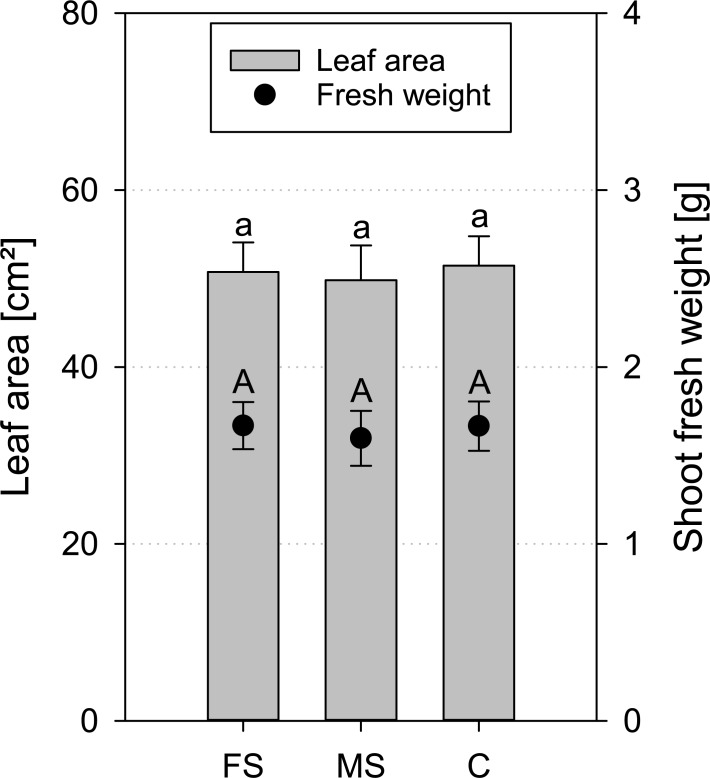
Mean leaf area and shoot fresh weight of *Hordeum vulgare* for frequent scanning (FS), moderate scanning (MS) and no scanning control treatment (C) at the end of the experiment (17 DAP); standard errors are given as error bars.

As for roots, no impact of X-ray scanning on shoot growth was detected. Neither leaf area nor shoot fresh weight was different for the scanned treatments in comparison to the control treatment (Fig 10).

## Discussion

Root growth development and the influence of cumulated X-ray dose on *Vicia faba* and *Hordeum vulgare*

X-ray CT enables studies of root growth development in a very high quality. The chosen scanning interval revealed essential steps of root system development of *Vicia faba* (Fig 1, S1–S5 Figs). At 4 DAP only the tap root had developed. At 8 DAP already several first order laterals had emerged and further initials could be recognised along the tap root. At 12 DAP first order laterals had developed and elongated along the whole length of the tap root; elongation of individual laterals could be accurately followed over time. The last scanning date (16 DAP) showed initiation of second order laterals (S4 and S5 Figs).

For *Hordeum vulgare* with most of the roots smaller than 0.5 mm (Fig 9), visualisation of root system development by X-ray CT was not possible with the chosen X-ray CT settings. These had to be the same for both plant species in order to enable a direct comparison of their susceptibility to a certain X-ray dose. Image quality for *Hordeum vulgare* could be improved by choosing smaller soil column diameter to achieve a higher resolution and by increasing the number of projections per scan. However, first this would reduce the soil volume available for root development and second this would increase scanning duration and therefore X-ray radiation dose. New algorithms like the one suggested in [25] can help to segment roots of *Hordeum vulgare* in general. But in the case of this study, assuring the comparability through identical scan settings was more important than an optimised detection of *Hordeum vulgare* roots.

In this study, we found a significant impact of cumulated X-ray radiation on plant development of *Vicia faba*. The influence was more pronounced for root traits, but also leaf area and shoot biomass was significantly smaller for plants exposed to X-rays in comparison to the control. Analysis with WinRHIZO revealed a clear difference of total root length between all three treatments of *Vicia faba*, but not for *Hordeum vulgare*. We have derived quantitative information about root length and number of first and second order laterals from the CT data for *Vicia faba*. Here, especially number and length of second order laterals was obviously affected by X-ray radiation.

Elongation of first order laterals was delayed by frequent application of X-ray CT. At MS the majority of first order laterals reached the container wall at 12 DAP. At FS, this was only the case at 16 DAP, indicated by the shorter orange segments in Fig 1, S5 Fig and S1 and S2 Videos.

Between 12 and 16 DAP most of the root growth in the moderate treatment occurs outside the scanned region of interest (ROI), which gives the high-dose treatment the chance to catch up with respect to root length densities within the ROI. We would not ascribe the observed behaviour to a bonsai effect due to confined growth in a limited pot volume, but simply to limiting our observation to the ROI.

The reduction in root elongation might be related to phytohormones like cytokinins, ethylene and especially auxin, essential for cell division and root elongation. Skoog [4] used different setups to study the effect of X-ray radiation on auxin and plant growth. Most experiments were performed with 900 kV and 3–4 mA, but with a layered filter consisting of lead, steel and aluminium to remove soft radiation. They either irradiated auxin directly in agar blocks or solutions, or seedlings and very young plants of *Vicia faba* and *Pisum sativum*. For example, at 50 Röntgen (600 kV and 3–4 mA) per minute, about 30 percent of uncovered auxin in agar blocks was destroyed after 30 seconds. The applied dose rate equals 0.44 Gy per minute. This is about 4 times higher compared to our study with less than 0.1 Gy per minute. Moreover, they could show that 30–40% of the auxin diffusing out from terminal buds and stem sections from *Vicia faba* were lost after irradiation of the plants in comparison to the control. In general they stated that by X-ray radiation, auxin is inactivated in solutions because of oxidation, that the formation of auxin is inhibited by moderate dosages, that decrease in growth is a function of dosage and that the mechanism of auxin transport is not affected. Given the fact, that auxin is not directly exposed to radiation in our case, as it is embedded in the root cells that are surrounded by soil that scatters and attenuates the radiation to some extent, it is likely that the influence is smaller. But still a considerable part of auxin may be destroyed by the accumulated dose of X-ray radiation during the scanning times. As auxin is also a key player in initiation of second order laterals, this is in line with the result that *Vicia* plants of both irradiated treatments had significantly less and shorter second order laterals (Fig 3) compared to the control. Unfortunately we could not find more recent literature investigating this auxin hypothesis for root development in a setting comparable to ours.

Evans [1] cited a work from Gray and Scholes [2], working with 143 R of X-rays. This equals about 1.3 Gy. In Gray and Scholes [2], a 80% reduction of the growth rate of the primary root of *Vicia faba* five days after irradiation was found. Moreover, it was shown that the reduction in root growth was the same if only the root tips received radiation. When the root tip was shielded, no reduction in root growth was found. This underlines the essential role of meristematic cells regarding sensitivity against X-ray radiation.

In particular young tissue with a high share of meristematic cells was sensitive to radiation. Cytological changes were observed in irradiated plant meristem cells [1]. Evans [1] reported that the mitotic cycle delay is transitory and full recovery was possible within 24 h. This recovery effect is also reported by [5, 6, 8]. Also our treatment with low scanning frequency had more time to recover compared to the treatment with high scanning frequency, but our experimental setup does not allow for separation of total dose and scan frequency effects. Evans [1] also stated that chromosome aberrations and influences on nuclear volume and DNA synthesis are crucial for growth inhibition. Similar results were achieved by Davidson [7], reporting viable atypical chromosome complements in cells of primary roots of *Vicia faba* by exposure to a dose of 600 r (equals 5.3 Gy), 24 hours after germination. These effects are expected to be even more pronounced, when the same plant is irradiated with a high frequency.

Zappala *et al*. [18] found no influence of X-ray radiation on root length and number of tips in rice (*Oryza sativa* spp. Azucena). X-ray settings were 110 kV, 320 μA, 0.2 mm Cu filter and a source to sample distance of 21.5 cm. This means lower kV, higher μA, thinner Cu filter and higher source to sample distance compared to our settings. Over a total of 9 scans of 73 minutes each, this resulted in a total dose of ~13 Gy per column (stated by the authors). This is about 5 Gy more than at the frequent scanning treatment in our study. The results of Zappala *et al*. [18] for *Oryza sativa* are similar to our results for *Hordeum vulgare*, showing no influence of X-ray radiation on root growth. The contrasting results for *Vicia faba* may be related to the very coarse and compact taproot system architecture of the dicotyledonous plant in comparison to the large and adventitious root systems of *Oryza sativa* and *Hordeum vulgare*, or cereals in general.

This is also confirmed by the results from Flavel *et al*. [10]. They reported that no effect of exposure to X-rays on *Triticum aestivum* L. cv. ‘Gregory’ was detected. They used 100 kV, 270 μA, a 0.5 mm Cu filter and very short scanning duration (4 minutes and 10 seconds per scan, maximum scanning duration was 20 minutes 50 seconds). Dose in Flavel *et al*. [10] cannot be quantified precisely, as information regarding distance between X-ray source and sample is missing but due to low energy settings and short scanning duration dose is estimated as rather low compared to our study.

Adventitious root systems have more root tips and meristematic cells than taproot systems. Therefore, statistically there are more cells available to respond to radiation. In turn, if a certain percentage of cells are affected by X-ray radiation, more meristematic cells in root tips stay intact to compensate and maintain root growth and by this plant development.

Moreover, plant age during the first exposure might have a strong relevance regarding sensitivity to radiation. Very often plants are only a few days old when they are placed in the tomograph for the first time (e.g. Gregory *et al*. [16]: “few days”; Hargreaves *et al*. [26]: 3–7 days). In our case, plants were scanned initially 4 days after planting. This is an early stage of development, but this is quite usual for many studies dealing with root development, measured by X-ray CT. In Zappala *et al*. [18], plants were older at the first scan (19 DAP). This might also be an additional reason why there was no influence on root growth of *Oryza sativa* in that case, but more research is needed to clarify the relevance of plant age.

The review of Zappala *et al*. [18] and in particular the comprehensive study of Johnson [3] covering 70 plant species provided first evidence for differences in sensitivity against radiation between species. For instance, Johnson [3] found changes of flowering time, change of flower number, varying average plant height at maturity for some species and stated a reduction in number and length of roots for at least one species (*Ricinus communis*). No general plant response can be derived, especially as some species were apparently completely unaffected by the X-ray radiation. Experimental conditions were not identical for all plant species and plant age varied between 8 and 74 days in the study of Johnson [3]. However, there is a need for more experiments with same settings comparing different plant species to increase understanding if the degree of sensitivity is a function of plant species (or families) root diameter, cell size, number of root tips or a combination of all parameters. This is especially true, as all studied plants in Johnson [3] were dicotyledonous, i.e. just representing one group of flowering plants, and even within this group considerable variation regarding sensitivity to radiation was found, e.g. a varying average plant height at maturity from -79% to +77%.

**Fig 11:**
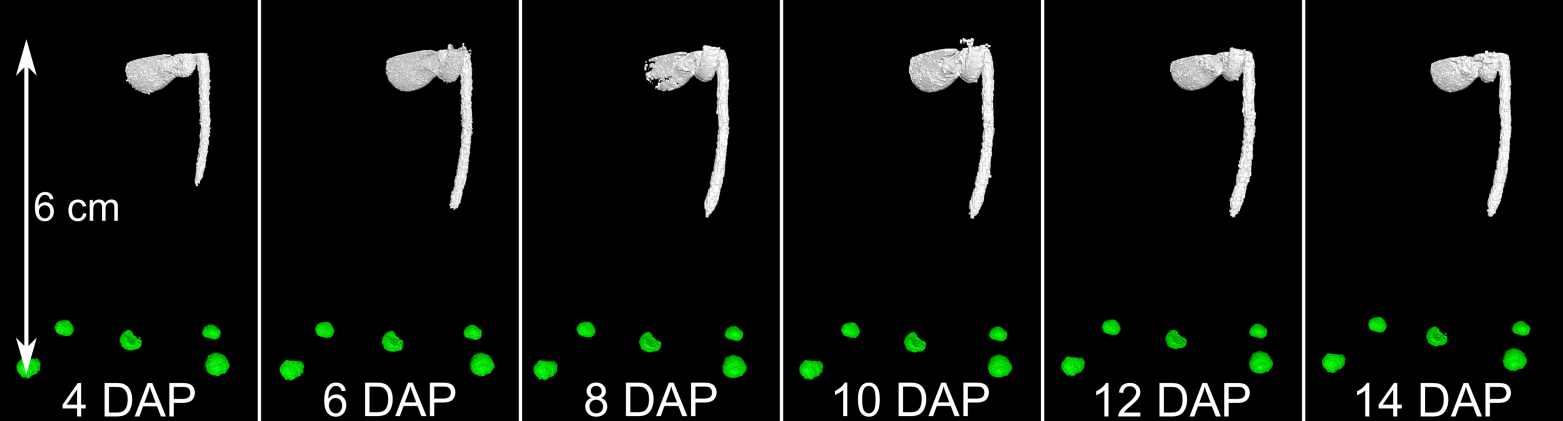
Time series of a taproot from *Vicia faba* visualised every second day by X-ray CT; scanning settings were chosen for best image quality; lethal influence on root tissue can be seen, as the root did not grow further within 14 days.

In one preliminary experiment focused on optimizing the CT parameters for best image quality and short scanning duration we applied a dose of >16 Gy per single scan, calculated with a 0.2 mm Al filter in the Rad Pro Calculator, as Al reduces dose the least among the available materials in the Rad Pro Calculator (further CT settings were: 810 μA, 140 kV, no filter, 8.5 minutes duration, same column size and similar substrate). In that case, *Vicia faba* plants died immediately after the first two scans (4, 8 DAP), having a tap root of less than 4 cm in length (Fig 11).

### Strengths and limitations of dose estimation

Estimation of cumulative dose through X-ray CT enables a certain comparability between different studies. This is important as a direct measurement of the cumulative dose received by the roots is not a standard procedure at the moment. This localised dose measurement in soil as a function of wall distance should be addressed and implemented in future research. Moreover, root segments receive location-dependent dose rates as the soil shields radiation. Roots directly at the container wall receive a dose rate comparable to the estimated maximum dose rate in air, whereas X-ray attenuation is highest in the centre of the soil column. This attenuation depends on bulk density, soil moisture and sample diameter. But since the CT scan settings are typically adjusted to these soil properties, the actual dose that reaches the centre of the column rather depends on the signal-to-noise ratio that one is willing to accept or able to achieve with a given detector panel and reconstruction software. In the course of the growth experiment the root explores the entire range of wall distances which impairs a good estimate of the actual, cumulative dose experienced by individual root segments and the whole root system, respectively.

Despite these shortcomings in assessing the actual dose, reporting estimates of the maximum dose in air has some merits, as it enables a quick comparison between X-ray CT studies. Yet, there are also some limitations and uncertainties in dose estimation, as the current version of the Rad Pro Calculator includes only the basic settings like voltage, current, distance between source and sample, filter material and filter thickness. Hence, not all specifics of the X-ray source are included. Moreover, effects like scattering, attenuation and beam hardening are not included. Furthermore we experienced changes within the calculation method in different versions of the calculator that resulted in different calculated doses. Finally, applying the same total dose as one single scan or several scans with lower dose per scan is not distinguished in the calculator. In clinical radiobiology the concept of “biologically equivalent doses” [27] of different scan frequencies is well established, but it is uncertain whether this also applies to root tissue.

### Recommendation on best practices

We have shown that *Vicia faba* and *Hordeum vulgare* have a very contrasting susceptibility to X-ray radiation. In our case all experimental conditions were kept the same and the plants also had the same age when irradiated. Further research is needed to investigate in detail why different plant species react differently to X-ray exposure. Moreover, additional studies are required to clarify the impact of e.g. plant age, soil properties, X-ray spectra and other parameters determined by the X-ray settings that may influence the susceptibility of plant roots to X-ray radiation.

In light of the 1) uncertainties in estimating the actual dose received by plants in pot experiments, 2) the different response of the two investigated species to the same dose and 3) the harmful irradiation effects in *Vicia faba* at relatively low dose for a typical growth experiment, we strongly recommend to always use an un-scanned control (i.e. only scanned once at the end) to adequately assess radiation effects. Moreover, the number of scans and dose per scan should be reported rather than the cumulated dose, to assess potential recovery effects. Finally, care should be taken to minimise the applied dose to the roots, especially when working with young tap rooted plants. This could also be done by shielding the part of the soil column that is not in the region of interest during the scan.

## Conclusion

We conclude that X-ray CT is a powerful method to observe root system development in the soil *in situ* with high quality of information. But there is a lack of information regarding the influence of X-ray radiation on root growth. As we could show, *Vicia faba* was affected significantly by X-rays, especially when scanned at a frequent temporal resolution (every 2^nd^ day in this case). In comparison, *Hordeum vulgare* showed no influence of X-ray radiation for the exact same sample conditions and scanning parameters. This information is very important to adjust experimental setups and scanning parameters in the future. When X-ray CT is used to study root dynamics as a response to e.g. nutrient availability, water distribution or soil mechanics it is essential to know about the effect by the method itself to enable reduction of this influence or to distinguish between method-associated reaction and dynamic response of the roots to soil-physical and chemical conditions. This is especially true for cumulative scanning setups for time-lapse analysis of root growth. Having an un-scanned control treatment is a viable option to estimate potential influence of the X-rays.

Further research is needed to investigate why different plant species have a different susceptibility to X-ray radiation and to elaborate on the impact of e.g. plant age and soil properties, as well as CT scanning parameters on the influence of X-rays on root growth.

## Supporting information

S1 Fig All root systems of *Vicia faba*, acquired by X-ray CT at 4 DAP. All root systems of *Vicia faba*, acquired by X-ray CT (representative 2D projections) at 4 DAP. Top row = frequent scanning (FS); bottom row = moderate scanning (MS). This was the first CT scan for both treatments.
(TIF)

S2 Fig All root systems of *Vicia faba*, acquired by X-ray CT at 8 DAP. All root systems of *Vicia faba*, acquired by X-ray CT (representative 2D projections) at 8 DAP. Top row = frequent scanning (FS); bottom row = moderate scanning (MS). All tap roots have grown below the region of interest and first order lateral roots have emerged.
(TIF)

S3 Fig All root systems of *Vicia faba*, acquired by X-ray CT at 12 DAP. All root systems of *Vicia faba*, acquired by X-ray CT (representative 2D projections) at 12 DAP. Top row = frequent scanning (FS); bottom row = moderate scanning (MS). First order lateral roots have elongated differently for both treatments.
(TIF)

S4 Fig All root systems of *Vicia faba*, acquired by X-ray CT at 16 DAP. All root systems of *Vicia faba*, acquired by X-ray CT (representative 2D projections) at 16 DAP. Top row = frequent scanning (FS); middle row = moderate scanning (MS); bottom row = control (only this one scan was performed). Second order lateral roots are much more pronounced at the control treatment.
(TIF)

S5 Fig Time series of root system development of all *Vicia faba* replicates, acquired by X-ray CT. Time series of root system development of all *Vicia faba* replicates, acquired by X-ray CT. Top row = frequent scanning (FS); bottom row = moderate scanning (MS). Root age is colour coded for 4 (black), 8 (green), 12 (orange) and 16 (purple) days after planting (DAP). Changes in position are also recorded; this is the reason for the green shade at the seed in b). Secondary thickening can also be seen by the purple shade around the upper part of both tap roots.
(TIF)

S6 Fig Washed-out root systems from *Hordeum vulgare* of all treatments. Washed-out root systems from *Hordeum vulgare* of all treatments at the end of the experiment (17 DAP). Top row = frequent scanning (FS); middle row = moderate scanning (MS); bottom row = control (C).
(TIF)

S1 Video Video of root grow of *Vicia faba* for one sample from the treatment frequent scanning (FS), acquired by X-ray CT. Video of root grow of *Vicia faba* for one sample from the treatment frequent scanning (FS), acquired by X-ray CT. Root age is colour coded for 4 (black), 8 (green), 12 (orange) and 16 (purple) days after planting (DAP). Changes in position are also recorded; this is the reason for the green shade at the seed in b). Secondary thickening can also be seen by the purple shade around the upper part of both tap roots.
(MP4)

S2 Video Video of root grow of *Vicia faba* for one sample from the treatment moderate scanning (MS), acquired by X-ray CT. Video of root grow of *Vicia faba* for one sample from the treatment moderate scanning (MS), acquired by X-ray CT. Root age is colour coded for 4 (black), 8 (green), 12 (orange) and 16 (purple) days after planting (DAP). Changes in position are also recorded; this is the reason for the green shade at the seed in b). Secondary thickening can also be seen by the purple shade around the upper part of both tap roots.
(MP4)

S1 Method Image processing of CT data for *Vicia faba*. (DOCX)
