# Supplementary figures and images for: How much is too much?—Influence of X-ray dose on root growth of faba bean (Vicia faba) and barley (Hordeum vulgare)
Source: PLoS One. 2018 Mar 26;13(3):e0193669. doi: 10.1371/journal.pone.0193669 (PMC5868774; doi:10.1371/journal.pone.0193669)

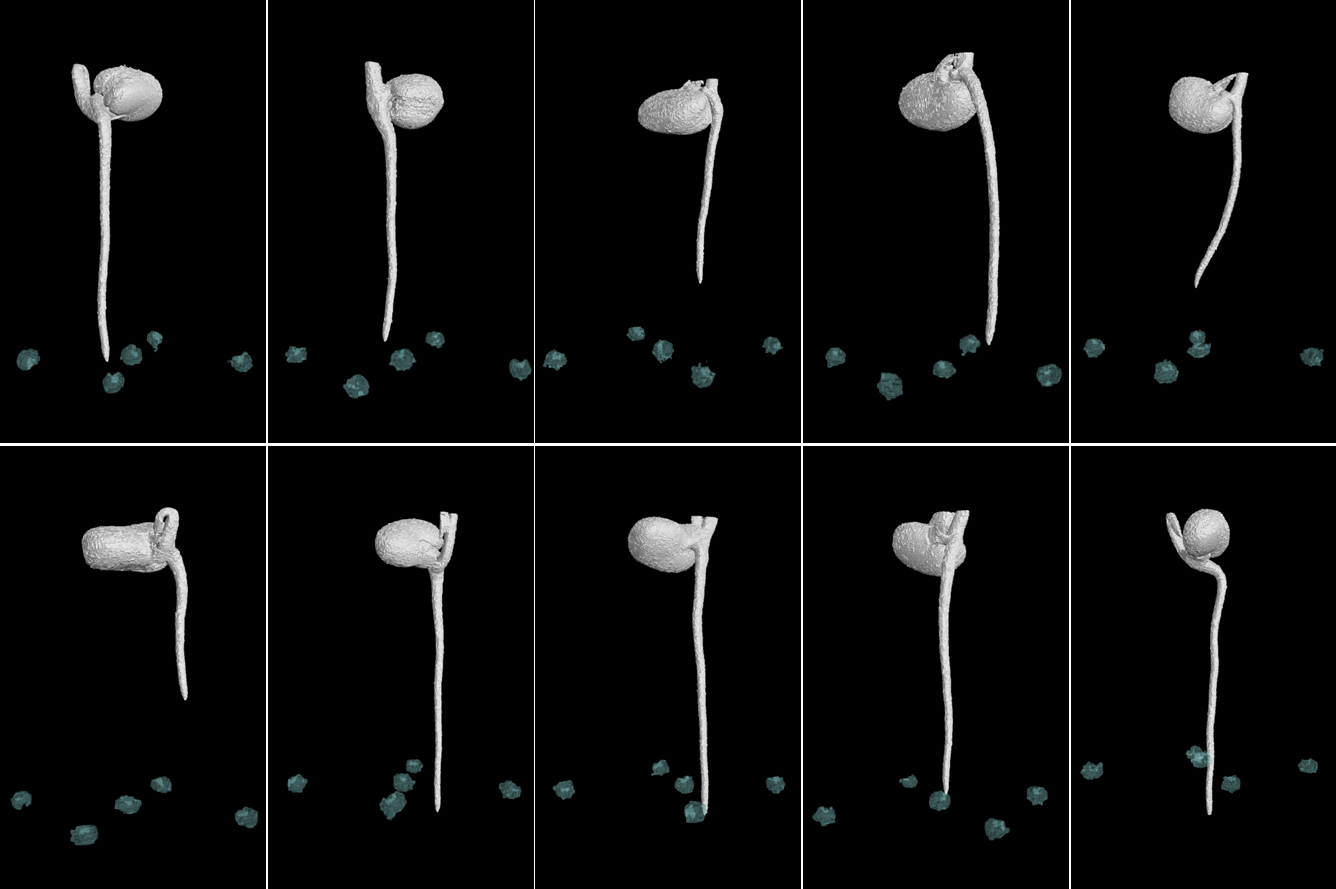

Supplement: S1 Fig — All root systems of Vicia faba, acquired by X-ray CT (representative 2D projections) at 4 DAP. Top row = frequent scanning (FS); bottom row = moderate scanning (MS). This was the first CT scan for both treatments. (TIF) [file pone.0193669.s001.tif]

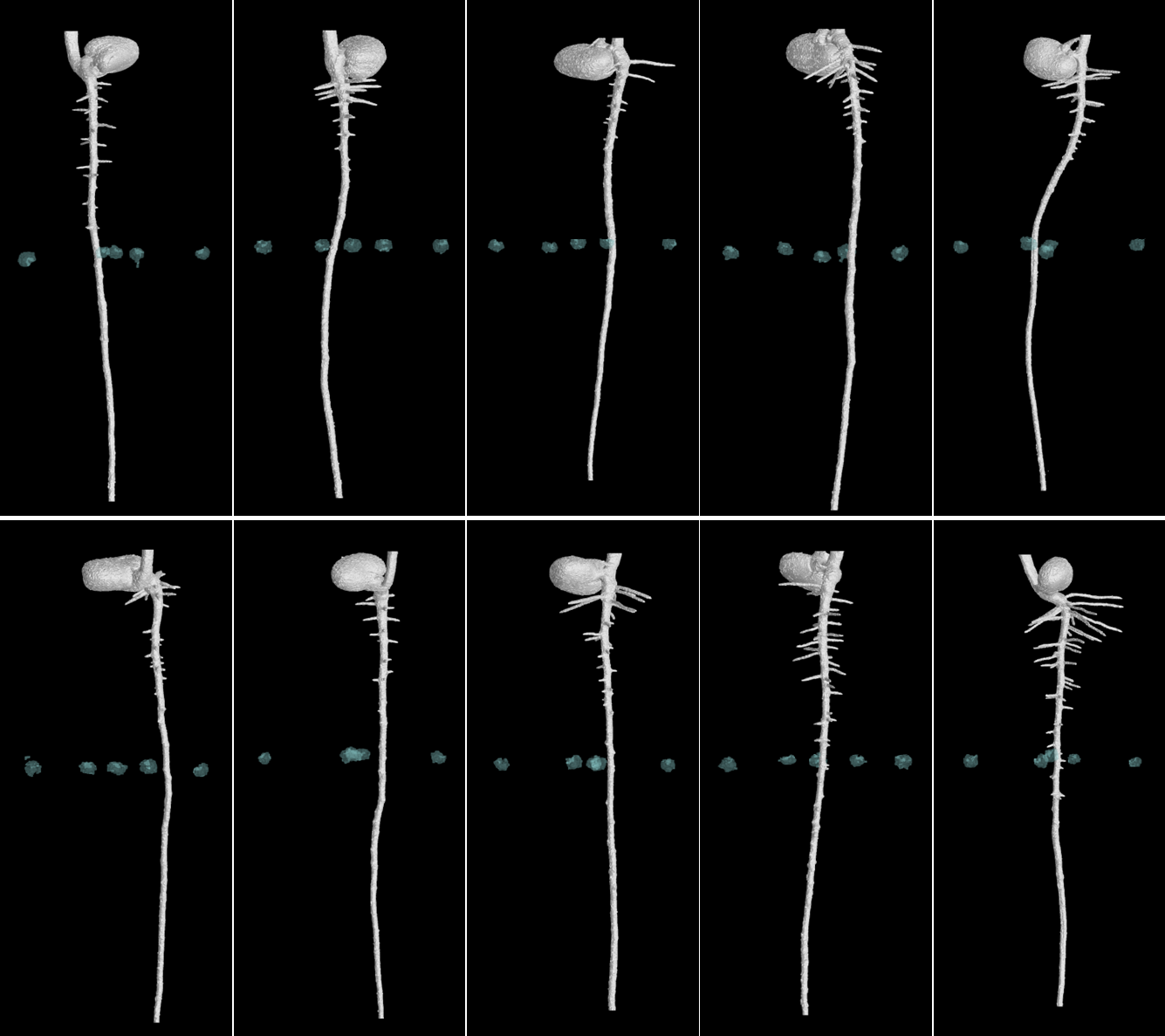

Supplement: S2 Fig — All root systems of Vicia faba, acquired by X-ray CT (representative 2D projections) at 8 DAP. Top row = frequent scanning (FS); bottom row = moderate scanning (MS). All tap roots have grown below the region of interest and first order lateral roots have emerged. (TIF) [file pone.0193669.s002.tif]

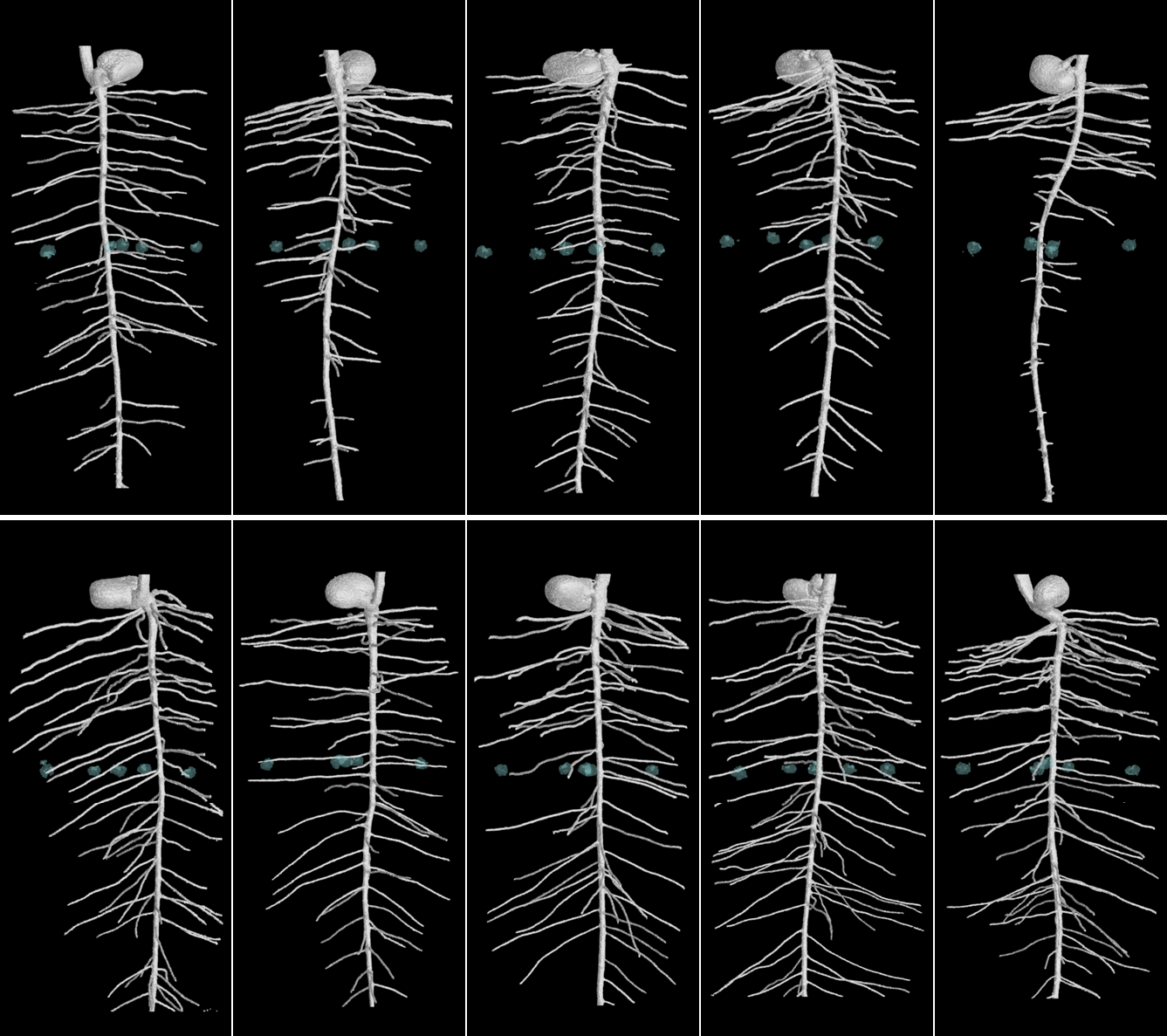

Supplement: S3 Fig — All root systems of Vicia faba, acquired by X-ray CT (representative 2D projections) at 12 DAP. Top row = frequent scanning (FS); bottom row = moderate scanning (MS). First order lateral roots have elongated differently for both treatments. (TIF) [file pone.0193669.s003.tif]

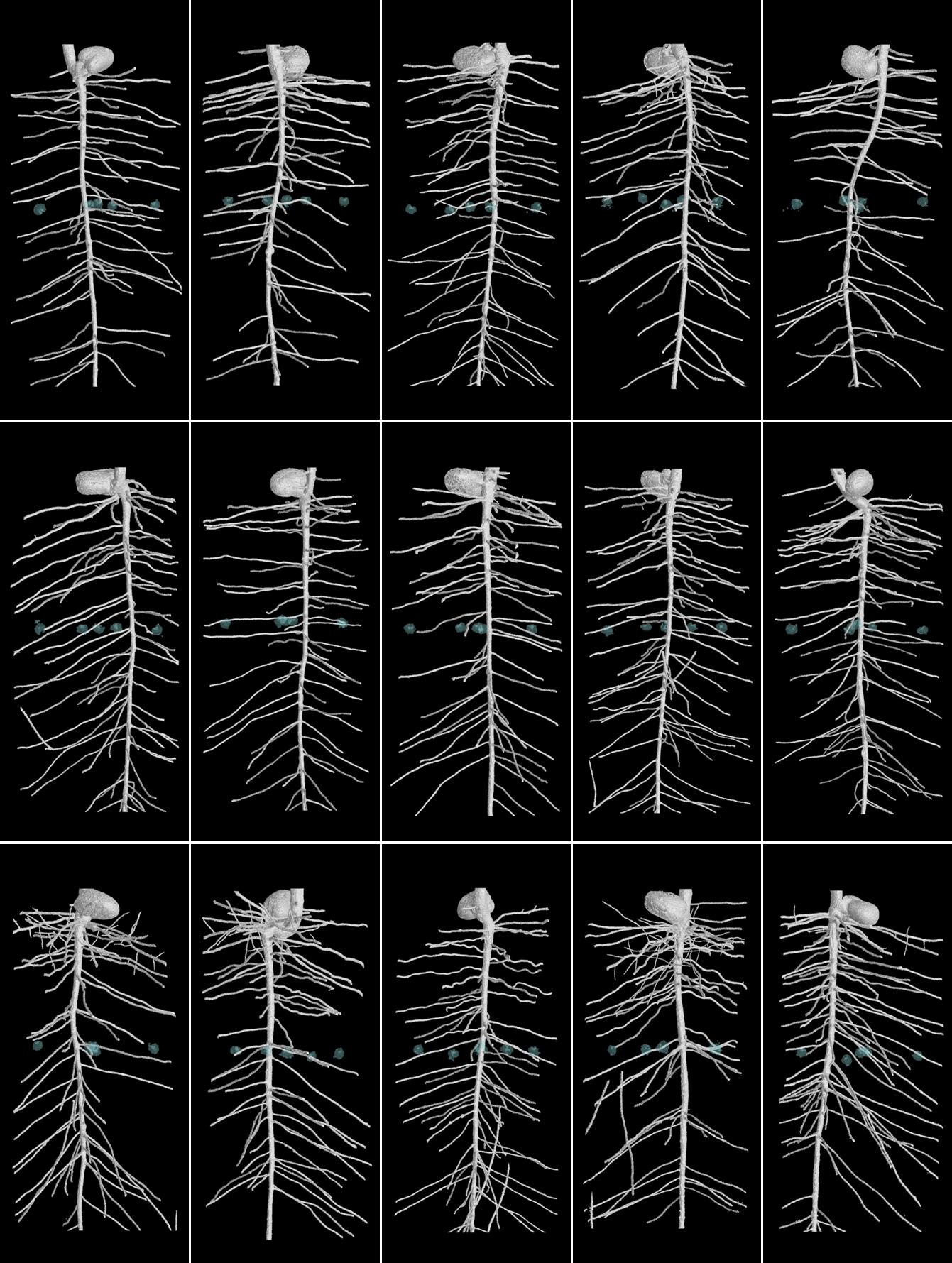

Supplement: S4 Fig — All root systems of Vicia faba, acquired by X-ray CT (representative 2D projections) at 16 DAP. Top row = frequent scanning (FS); middle row = moderate scanning (MS); bottom row = control (only this one scan was performed). Second order lateral roots are much more pronounced at the control treatment. (TIF) [file pone.0193669.s004.tif]

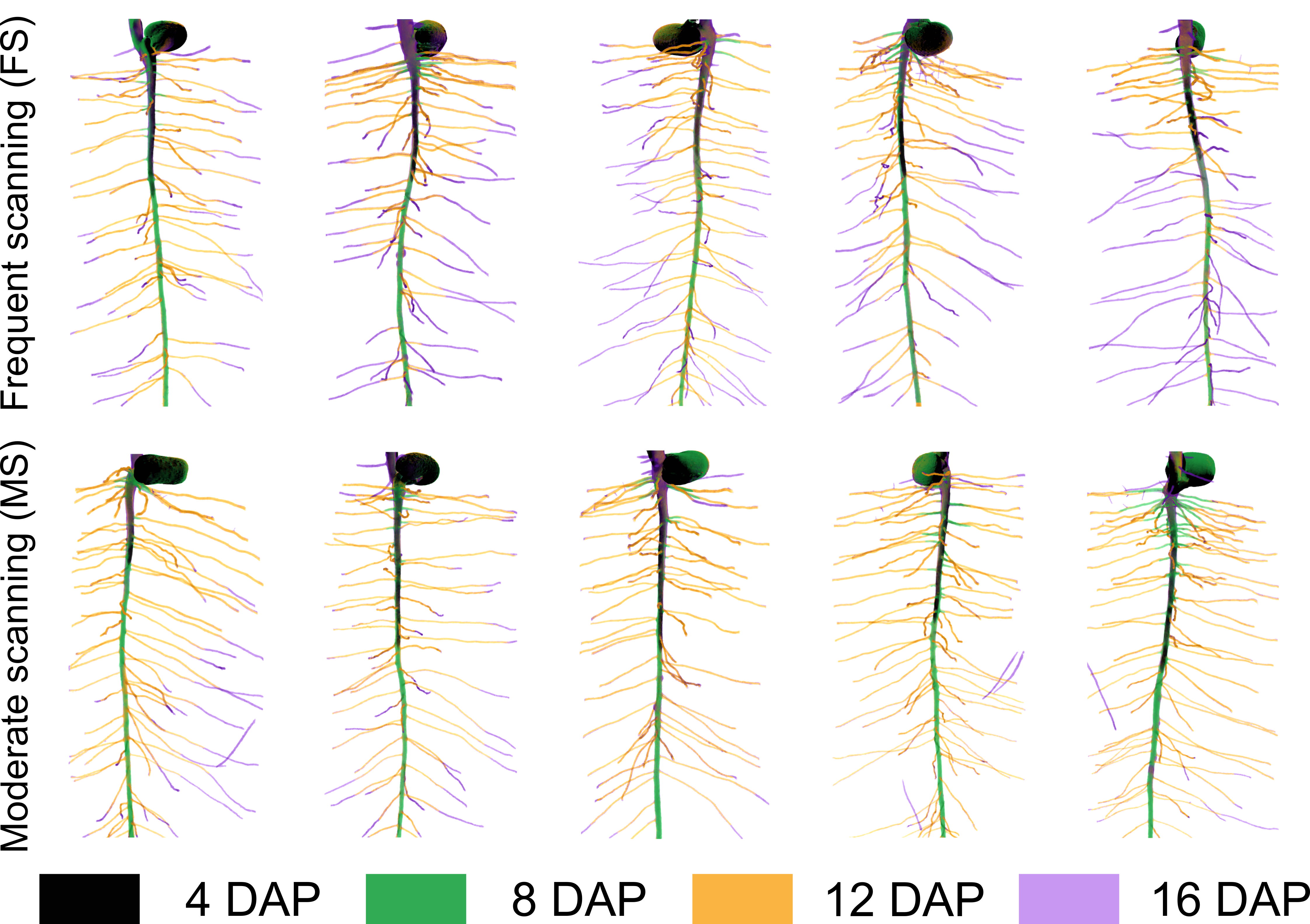

Supplement: S5 Fig — Time series of root system development of all Vicia faba replicates, acquired by X-ray CT. Top row = frequent scanning (FS); bottom row = moderate scanning (MS). Root age is colour coded for 4 (black), 8 (green), 12 (orange) and 16 (purple) days after planting (DAP). Changes in position are also recorded; this is the reason for the green shade at the seed in b). Secondary thickening can also be seen by the purple shade around the upper part of both tap roots. (TIF) [file pone.0193669.s005.tif]

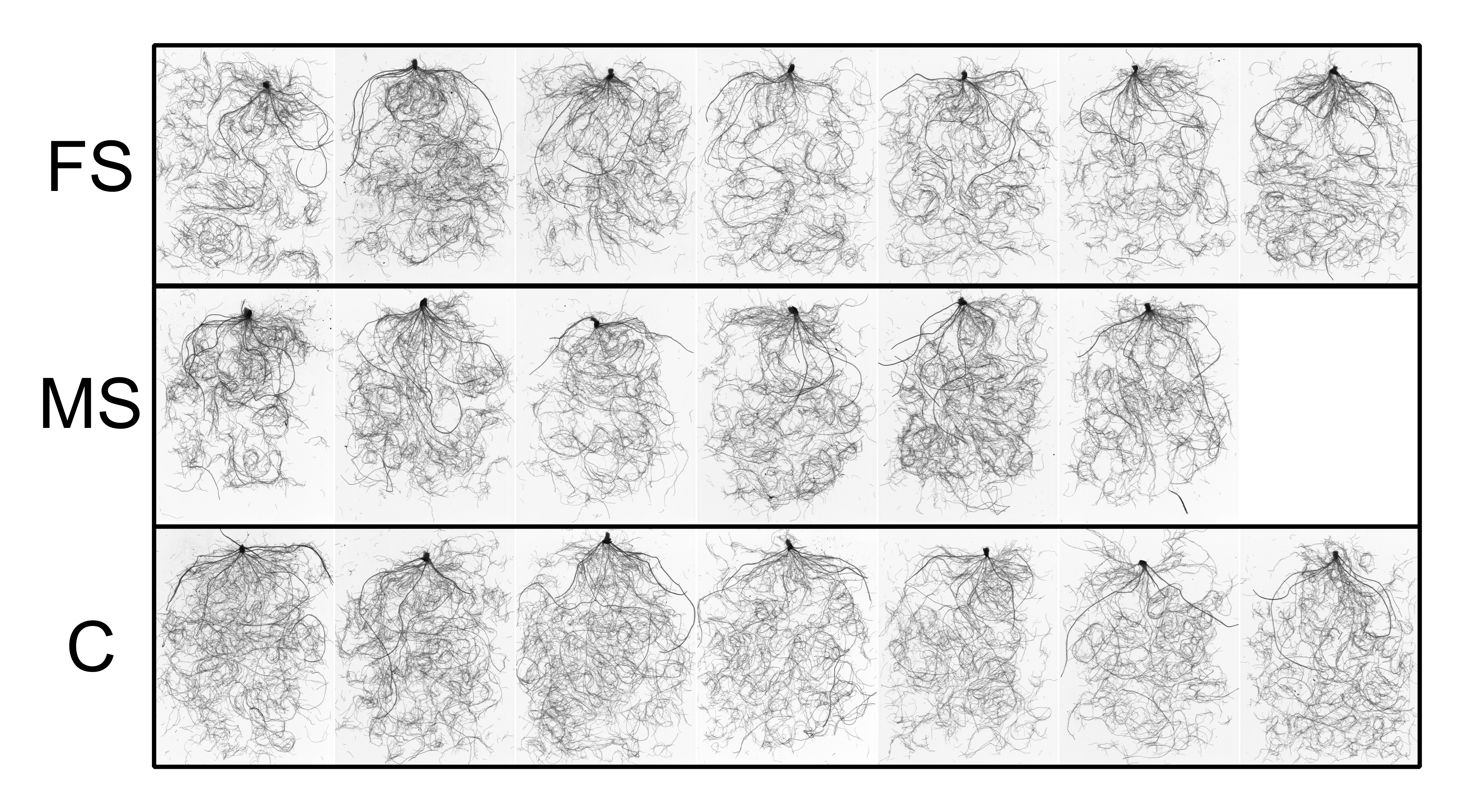

Supplement: S6 Fig — Washed-out root systems from Hordeum vulgare of all treatments at the end of the experiment (17 DAP). Top row = frequent scanning (FS); middle row = moderate scanning (MS); bottom row = control (C). (TIF) [file pone.0193669.s006.tif]
