# Supplementary material for: How much is too much?—Influence of X-ray dose on root growth of faba bean (Vicia faba) and barley (Hordeum vulgare)
Source: PLoS One. 2018 Mar 26;13(3):e0193669. doi: 10.1371/journal.pone.0193669 (PMC5868774; doi:10.1371/journal.pone.0193669)
Supplement: S1 Method — (DOCX) [file pone.0193669.s009.docx]

**S1 Method. Image processing of CT data for *Vicia faba*.**

For *Vicia faba*, a detailed analysis of CT-images was conducted. Raw images were filtered with a 3D Gauss-filter with kernel size 5 to reduce image noise. The Gauss-filter was chosen, as it is fast, robust and sufficient enough for the high diameters of the *Vicia faba* roots. Root systems were segmented with dynamic semi-automated region growing in VG Studio Max 2.1. The segmented root volumes were exported as .RAW files from VG Studio and processed with Fiji [1], a distribution of ImageJ [2]. Root volumes from the top and bottom CT-images were merged (either with pairwise stitching or manually by canvas size, translate and concatenate), binarized and filtered (3D Median-filter kernel size 5) to smoothen the outer surface to prevent that surface roughness is detected as false branches in the next step. Based on the idea of Flavel *et al.* (2012), root volumes were skeletonized and analyzed with the Fiji plugin ‘BoneJ’ [3]. The resulting information about all detected ‘branches’ (in the following denoted as segments) was used to distinguish the tap root from first and second order laterals by the three-dimensional extension of every segment and their position in the 3d space. False segments were discarded, when either of the following criteria was met: (a) length < 25 voxel (= 1 mm), (b) overlay of the complete detected segments with the extension of the seed from *Vicia faba* (detected manually) or (c) ratio between geodesic length and Euclidian distance > 2. All queries and summations are performed in Excel.

Segments with maximum extension in direction of the z-axis are referred as taproot. The sum of all segments defined as tap root results in the total tap root length. This is true for the first three points in time 4 DAP, 8 DAP and 12 DAP, as only taproots and first order laterals (for 8 and 12 DAP) existed. Later on, this procedure would overestimate the length of the tap root, as most of the second order laterals were also oriented vertically. Hence, the corresponding coordinates in x- and y-direction were used as additional information for proper assignment. This is valuable, as it refers as a query to detect lateral segments having a connection with these tap root coordinates. These junction points enable the detection of the number of first order laterals emerging from the tap root. It is important to note that in the skeletonize algorithm in Fiji, every segment is defined from one junction to the next junction or to the end point of the segment. Therefore, number of laterals would be overestimated, if every single segment with mainly horizontal expansion would have been counted as a single lateral root. For 16 DAP, the coordinates from 12 DAP were used and 100 voxels were added for the maximum extension and subtracted for the minimum extension of the tap root in both, x- and y-direction, respectively. This is necessary, as ImageJ works with relative coordinates, depending on the total spatial expansion of the stack. Therefore, coordinates are not exactly the same for 12 and 16 DAP. Because of the widening of the tap root coordinates, the detection of number of first order laterals is distorted for 16 DAP, as too many segments would be defined as having a junction to the tap root. Hence, number of first order laterals is not analyzed for 16 DAP. Moreover, no crucial further increase was detected visually after 12 DAP. Values for taproot extension at control treatment were selected manually within Fiji, as no information from earlier points in time exist. Second order laterals were detected as being vertically oriented, but not defined as tap root by comparison with the coordinates of the known tap root from 12 DAP. By this, some small fragments and some second order laterals are not captured, in case they were to short or oriented more horizontally, but still this approach leads to a very high level of detail and results are promising and plausible. Number of second order laterals can be verified by eye, because of the small and compact root system of *Vicia faba* and results match largely, with only few underestimations. In summary, the combination of root segmentation by a region growing algorithm with the analysis of root architecture by a skeleton analysis is not yet fully automatic, but results in a robust estimation of the morphological changes during root growth.

CT-images of segmented roots of the same plant at different time steps are not spatially aligned, since it is impossible to relocate the exact position and orientation of the columns at each scanning date with microscopic precision. To overlay all time steps in one time series (Fig 1, S5 Fig, S1 Video and S2 Video) we registered the images with the software elastix [4] through an Euler transform (translation and rotation). An optimal registration was achieved by simultaneously maximizing the correlation coefficient between co-located voxels and minimizing the Euclidean distances between manually chosen corresponding points in both images. Convergences was accelerated with image pyramids, that allow for fast optimization at a coarse resolution and subsequent refinement at higher resolution. More information about the chosen parameters can be found in [5].

References

1. Schindelin J, Arganda-Carreras I, Frise E, Kaynig V, Longair M, Pietzsch T, et al. Fiji: an open-source platform for biological-image analysis. Nature methods. 2012;9(7):676-82.

2. Schneider CA, Rasband WS, Eliceiri KW. NIH Image to ImageJ: 25 years of image analysis. Nat methods. 2012;9(7):671-5.

3. Doube M, Kłosowski MM, Arganda-Carreras I, Cordelières FP, Dougherty RP, Jackson JS, et al. BoneJ: free and extensible bone image analysis in ImageJ. Bone. 2010;47(6):1076-9.

4. Klein S, Staring M, Murphy K, Viergever MA, Pluim JP. Elastix: a toolbox for intensity-based medical image registration. IEEE transactions on medical imaging. 2010;29(1):196-205.

5. Schlüter S, Leuther F, Vogler S, Vogel H-J. X-ray microtomography analysis of soil structure deformation caused by centrifugation. Solid Earth. 2016;7(1):129.
